# Thoughts on Scientific Evidence in the COVID-19 Era

**DOI:** 10.1017/dmp.2020.213

**Published:** 2020-06-24

**Authors:** Giacomo Mugnai, Carla Paolini, Claudio Bilato

**Affiliations:** Division of Cardiology, West Vicenza General Hospitals, Arzignano (Vicenza), Italy

**Keywords:** mass media, scientific evidence, social media

The number of individuals infected by the severe acute respiratory syndrome coronavirus 2 (SARS-CoV-2) is dramatically increasing worldwide, and the SARS-CoV-2-related disease (COVID-19) is spreading broadly over all the continents.^1^ Collecting as much experimental and clinical data as possible has become, therefore, a major challenge for the international scientific community in order to promptly provide the most appropriate and effective strategies for the management and the treatment of this disease. So far, most of the evidence includes clinical pictures, complications, and therapies resulting frequently from small, unblinded, non-randomized reports. How much is the scientific value of these observations? Is the scientific evidence enough to drive daily clinical practice? Or should we treat these studies only as hypothesis-generating experiments?

There are no doubts that the COVID-19 pandemic is a worldwide emergency, which requires all possible available information. On the other hand, caution should be applied in generalizing preliminary (although potentially useful) observations as true scientific evidence.

In the last few weeks, many people, including politicians and even scientists, have enthusiastically called for several “miraculous” drugs, as a sort of the “Holy Grail” able to defeat the SARS-CoV-2. Every day, the newspapers report the efficacy of new drugs for the COVID-19 treatment, based on empirical, off-label tests of existing drugs in small non-randomized groups or on *in vitro* experiments of new molecules. On the contrary, avoiding sensationalism and false beliefs in the public opinion should be a priority for the medical community and the health agencies and organizations.

A few years ago, in Italy, several clinical trials were run by the National Health System on the “Di Bella” therapy for cancer,^2^ more because of the pressure of public opinion than objective preclinical observations. Currently, a clinical trial on the Japanese drug, favipiravir, has been called on the basis of a social media-posted video, ignoring that all the drugs need to be followed with specific preclinical and clinical steps before definitive approval.

Medical history is full of examples of drugs with harmful effects discovered after being largely used, such as thalidomide,^3^ diethylstilbestrol,^4^ and rofecoxib.^5^ Hydroxychloroquine has recently been used as first-line therapy in COVID-19 patients and also proposed as a “preventive” agent,^6^ but definitive evidence on its efficacy and safety (eg, QT prolongation) is missing, and, recently, the US Food and Drug Administration cautioned against hydroxychloroquine use in non-hospitalized COVID-19 patients, due to a number of side effects, including serious heart rhythm problems that can be life-threatening.^7^

It is true that the ongoing pandemic needs timely and prompt answers, so, what do we do while we wait? We may upgrade the debate on COVID-19 topics among colleagues at “high intensity” and quality level, and we may promote dedicated groups on social networks, restricted to physicians and other health care professionals, in order to share personal experiences, to ask for medical advice, and to trade case reports. We may also take some shortcuts with a “calculated risk” or, better, encourage to join the global efforts in developing the vaccine against the SARS-CoV-2. In any case, we have always to remember that, when scientific evidences are still weak, the risk of dispensing ineffective and unsafe drugs is “just around the corner.”
